# Young people’s explanations for the decline in youth drinking in England

**DOI:** 10.1186/s12889-022-14760-y

**Published:** 2023-02-28

**Authors:** Victoria Whitaker, Penny Curtis, Hannah Fairbrother, Melissa Oldham, John Holmes

**Affiliations:** 1 School of Health and Related Research, University of Sheffield, Sheffield, UK; 2 Health Sciences School, University of Sheffield, Sheffield, UK; 3 Behavioural Sciences and Health, University College London, London, UK

**Keywords:** Alcohol, Qualitative, Adolescent, Child, Health, Trends, Culture, Risk

## Abstract

**Background:**

Youth alcohol consumption has fallen markedly over the last twenty years in England. This paper explores the drivers of the decline from the perspectives of young people.

**Methods:**

The study used two methods in a convergent triangulation design. We undertook 38 individual or group qualitative interviews with 96 participants in various educational contexts in England. An online survey of 547 young people in England, was also conducted. Participants were aged between 12–19 years. For both data sources, participants were asked why they thought youth alcohol drinking might be in decline. Analysis of interview data was both deductive and inductive, guided by a thematic approach. Content analysis of survey responses further refined these themes and indicated their prevalence within a larger sample.

**Results:**

The research identified eight key themes that young people used to explain the decline in youth drinking: The potential for alcohol-related harm; Contemporary youth cultures and places of socialisation; The affordability of alcohol; Displacement of alcohol by other substances; Access and the regulatory environment; Disputing the decline; Future Orientations; and Parenting and the home environment. Heterogeneity in the experiences and perspectives of different groups of young people was evident, particularly in relation to age, gender, and socio-economic position.

**Conclusions:**

Young people’s explanations for the decline in youth drinking in England aligned well with those generated by researchers and commentators in prior literature. Our findings suggest that changing practices of socialisation, decreased alcohol affordability and changed attitudes toward risk and self-governance may be key explanations.

**Supplementary Information:**

The online version contains supplementary material available at 10.1186/s12889-022-14760-y.

## Background

Youth drinking is in decline in many high-income countries. This global trend manifests in terms of delayed age of initiation of drinking, and reductions in the volume and frequency of alcohol consumption [1–4]. While approximately synchronous national declines in youth drinking are evident in a number of high-income countries, there is substantial variation between countries in these declines [5–7]. Although youth alcohol consumption in England was high at the turn of the millennium, relative to international counterparts, England has nonetheless seen particularly sharp declines in youth alcohol drinking, especially amongst boys [4].

A number of potential drivers have been proposed for these changes. These include policy initiatives targeting underage purchasing [8, 9], the role of migration from countries with abstemious attitudes to alcohol [9–11], economic factors that have made alcohol less affordable to young people [9, 12, 13], changes in prevailing social norms, particularly young people’s conscientiousness with regard to education [6, 14, 15], changes towards more authoritative and warm parenting relationships [16–21], drug substitution – in particular toward the use of cannabis [22–24], greater health consciousness amongst young people [9, 25, 26] and multiple mechanisms related to the proliferation of digital and internet-based technologies, including changes in the spatio-temporal structure of young people’s lives [9, 27]. However, reviews have concluded that there remains insufficient evidence in favour of any of these explanations as a key driver of the decline in youth drinking [5, 9, 25]. Instead, researchers have increasingly sought more complex explanations that draw on the interaction of multiple factors or the extent of change is moderated by country-level characteristics [28, 29], although these remain untested.

Qualitative studies echo many of these findings. For example, Torronen et al. [30] suggest, among other explanations, that drinking is no longer a key expression of normative masculinity, that social media deters drinking by facilitating surveillance of drunken behaviour while also provide socialising opportunities that do not involve drinking, and that a social trend towards healthiness has required a broad reorganisation of some young people’s habits and practices [31–33]. Despite some accounts suggesting that non-drinking is achievable due its normality within some age and peer groups [34], a separate literature also describes the complex strategies that many young people employ to achieve light drinking or abstinence. These include discrete management of consumption (e.g. pouring away half-finished drinks or pretending a non-alcoholic drink contains alcohol), claiming to be ‘taking a night off’ or actually having short- or long-term periods of abstinence while retaining the possibility of drinking in future [35, 36]. Such strategies suggest that light- or non-drinking young people must still negotiate social tensions around alcohol consumption despite the decline in youth drinking, although successful negotiation of these tension may reinforce positive self-perceptions of autonomy, individuality and authenticity [37].

In this paper, we draw upon the social studies of childhood to inform a child-centred approach, and draw on two methods to explore young people’s own ideas about what might be driving the decline in youth alcohol consumption. This conceptual approach [38] is concerned with understanding “how children learn about the social world, the sense they make of it and the ways in which their experiential sense-making might shape the things they choose to do, the opinions they express and the perspectives on the world that they come to embrace and embody” ([38]:1). We take care to avoid the positioning of young people as somehow marginal to society and, instead, proceed from a standpoint that acknowledges that young people are active participants within society [39]. As social beings, young people learn about the world in which they live, and their place within it, through interactions with others. Our conceptual approach thus necessarily highlights the need to appreciate young people’s locatedness within networks of familial and extra-familial relationships of dependence, independence and interdependence [40].

Moreover, in grounding our research approach within the social studies of childhood, we also pay attention to the views of young people as individuals: “differentiated not only by gender, ethnicity, age or health status but also by the different and particular circumstances of their own biographies” ([38]:1). Reflecting this stance, we therefore present, in this paper, a child-centred account, articulated through the voices of individual young people and presented in verbatim quotes. This allows for an understanding of how young people themselves may be contributing to creating and sustaining the declines in alcohol consumption, rather than analysing these as phenomena happening to them and driven by forces external to them.

## Methods

Data were generated in two phases between November 2018 and July 2019 within a convergent triangulation design [41]. This aimed to cross-validate young people’s perspectives on the decline in youth drinking using different data sources. In Phase 1, we undertook face-to-face discussions with young people in schools and other educational establishments. In Phase 2, we conducted an online survey to estimate the prevalence of different perspectives on the decline in youth drinking. As the survey used free-text responses, it also provided additional qualitative data to support the Phase 1 data. Triangulation took place during data analysis to align key themes across data sources, and then by comparing the prevalence of themes in the Phase 2 survey to their prominence and participants’ perceptions of their importance within the Phase 1 qualitative data.

Two age cohorts of young people were recruited: cohort 1 (C1) aged 12–15 and cohort 2 (C2) aged 16–19. These age cohorts were defined with reference to a larger project on the decline in youth drinking, of which this study is one component [42]. The data was collected in England, where the minimum legal purchase age for alcohol is 18. Although there is no prohibition on serving alcohol to anyone aged over five in a private home and over-16 s can consume alcohol (but not spirits) in a pub if it is served with a meal and they are accompanied by an adult, underage drinking is generally understood to mean drinking before age 18. We therefore treat such references in our data accordingly.

### Phase one methods

#### Recruitment

Ninety-six young people (Supplementary Table 1) were recruited from 5 socio-economically and geographically contrasting schools (two urban affluent, one urban deprived and two rural), two further education (FE) colleges and a university. All were within or close to a single city in the North of England.

Schools and FE colleges were identified by reference to the proportion of young people in each school in receipt of government financial support for school meals [43]. Rates of eligibility for free school meals are reported as percentage ranges to protect confidentiality (Table 1). Although free school meal data was absent for one of the FE colleges, it was chosen as a research site as it was situated in a highly deprived local authority area. The relative rurality of schools was determined through assessment of the distance between the school site and the city centre, and local area knowledge (these characteristics are not reported to protect the confidentiality of participating schools). University students were identified using an internal university email distribution list.

**Table 1:** Sample characteristics; educational contexts

**Educational context**	**Cohort**	Number of interviewees	Urbanity	% of school’s pupils eligible for free school meals
School 1	1 & 2	24	Urban	0–10%
School 2	2	5	Urban	0–10%
School 3	1 & 2	25	Urban	31–40%
School 4	1 & 2	19	Rural	21–30%
School 5	2	11	Rural	31–40%
Further Education College 1	2	3	Urban	0–10%
Further Education College 2	2	3	Urban	Unknown
University	2	7	Urban	Not applicable

Recruitment was planned to take place in three schools (1 affluent, 1 deprived and 1 rural) As the first rural school admitted young people up to the age of 16 only, a second rural school, enrolling young people up to the age of 18, was also included. Following school-based recruitment and data collection, an under-representation of older, affluent school pupils was noted. Five additional C2 pupils from a second, urban affluent school were therefore recruited.

Information sheets were distributed prior to interviews, via email. For participants aged under 16, separate child and parent information sheets and parental consent forms were provided to schools, and distributed to both parents and young people.

All interviews were scheduled at the convenience of the educational institution and took place in either educational establishments or participants’ homes. On the day of the interview, the researcher recapped the information sheet and provided an opportunity to ask questions. All participants provided written consent and were advised that they could withdraw their consent to participate at any point. Written parental consent for young people aged under 16 was a prerequisite of participation. Participants were remunerated for their time with shopping vouchers and a small financial reward was offered to each participating school.

#### Data generation

Data were generated in 38 semi-structured, interviews. Participants had the option to participate individually or in friendship groups – according to their preferences. There was one individual interview with a university student and all other interviews were conducted in small friendship-groups, comprising between two and four young people. Friendship group interviews are argued to help young people to feel comfortable in sharing their views [44]. Interviews in schools (*n* = 31) and colleges (*n* = 4) were constrained by lesson duration and typically lasted between 40 and 60 min. The remaining interviews were not time-limited in this way and lasted up to an hour and a half. Interviews explored alcohol within the context of peer and family relationships, and in relation to other consumption and health practices. While interviews employed a range of creative, participatory methods (to be reported in a future publication), this paper reports young people’s spontaneous responses to the ‘mini-debate’ question, “why do you think young people today are drinking less alcohol?”.

#### Data analysis

Anonymisation of interviews took place at the point of transcription. Analysis was aided by the qualitative analysis software, NVivo 12. Given the congruence between the reasons for the decline that young people asserted in interviews and those discussed in the extant literature, debate data were deductively coded under the following headings: ‘Disputing the decline’, ‘Drug substitution’, ‘Digital technology’, Difficulty of access’, ‘Economic reasons’, ‘Parenting’, ‘Awareness of health risks’ and ‘Other’. Data recorded under each code was further sub-divided by educational context and age group. Members of the research team independently reviewed a sub-set of codes to facilitate an inductive, thematic analysis that facilitated the deduction of eight themes [45]. These initial eight themes were further refined via triangulation with the phase two data (see below). Final thematic headings are described at the start of the results section.

### Phase two methods

#### Recruitment

Survey participants were recruited using targeted, paid-for advertisements on both Facebook and Instagram. This approach is responsive to the dynamic patterns of young people’s social media use [46]. The age range and geographical reach of the adverts aligned with Phase One of the methods to ensure a comparable sample. Participants provided consent before they were able to access the survey questions. Completion of the survey entered participants into a prize draw for a shopping voucher.

#### Data generation

Participants completed an online survey, developed and administered using Qualtrics. The survey included two demographic questions (age and gender) followed by a free text question asking why the respondent thought youth drinking was in decline.

To reduce the likelihood that respondents would falsely specify their age in order to be eligible for the prize draw [47], we did not disclose the study’s age range to survey participants. Although we included all respondents in the prize draw, data from participants outside of the 12–19 age range were not included in our analyses. To minimise the time commitment required to complete the survey and promote engagement, we restricted the collection of demographic data to the factors noted (gender and age).

The online survey was completed over a period of two weeks, by 547 young people aged 12–19 years (Table 2).

**Table 2:** Survey results; number of survey respondents by cohort and gender

	**Male**	**Female**	**Prefer not to say**	**Total**
C1	78	129	2	209
C2	121	212	5	338
Total	199	341	7	547

#### Data analysis

Survey data were first stratified by age and gender before undertaking an initial reading of the free-text responses. Respondents typically provided a single sentence in response to the free-text question, but multiple reasons for the decline in youth drinking were often given.

The initial reading of the free-text responses suggested broad convergence with the eight themes identified in the interviews, but also enabled refinement of the initial themes as part of the triangulation process. For example, the concept of future orientations was developed to include notions of futurity related to work, not solely higher education. This refinement generated a common set of themes from phases one and two that structure our reporting of findings and comparison across data sources.

To assess the prevalence of each theme within the phase two dataset, we undertook a content analysis to code each of the survey responses to one of the eight themes and calculated the proportion of respondents citing each theme. Reflecting the multiple reasons that young people often gave in their free-text responses to the survey, individuals’ responses could be coded to multiple themes. Content analysis was undertaken by VW and PC, any coding disagreement was resolved by discussion. (Supplementary Table 2).

In the results below, we triangulate the phases one and two data by comparing the prevalence of themes in the survey with their prominence in the qualitative data and by using the free-text responses in the survey as qualitative data to provide support for or contrast with themes from the interviews. The information about the speaker provided after quotes is more detailed for phase one interviewees than phase two survey respondents, reflecting the data collected.

## Results

The research identified eight key themes that young people used to explain the decline in youth drinking: The research identified eight key themes that young people used to explain the decline in youth drinking: The potential for alcohol-related harm; Contemporary youth cultures and places of socialisation; The affordability of alcohol; Displacement of alcohol by other substances; Access and the regulatory environment; Disputing the decline; Future Orientations; and Parenting and the home environment.

**Fig. 1:**
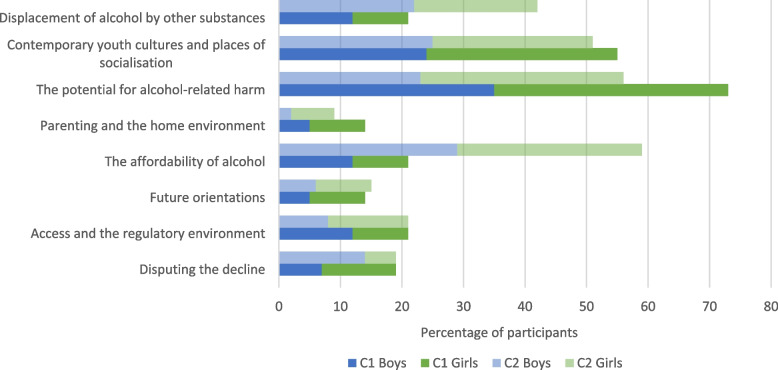
Percentage of respondents citing explanations for the decline in youth drinking by cohort and gender

The most commonly cited reasons for the decline in youth drinking identified by survey respondents fell under the themes ‘the potential for alcohol related harm’ (32%), contemporary youth cultures and places of socialisation (27%), and ‘affordability’ (22%). Young people were least likely to provide responses categorised as ‘parenting’ (6%), ‘future orientations’ (8%), or ‘disputing the decline’ (9%). There were some notable differences in responses between age cohorts, with largest disparities seen in relation to the themes of: affordability (C2 = 29%; C1 = 10%) and displacement of alcohol by other substances (C2 = 21%; C1 = 10%) (Supplementary Table 2; Fig. 1).

In the following sections we use the interview data to discuss each theme, starting with those cited by most respondents in the survey, and then use the survey data to provide additional insight.

### The potential for alcohol-related harm

Young people consistently noted a diversity of risks associated with the consumption of alcohol. There was broad understanding of the chronic health harms associated with alcohol consumption, including liver disease, cancer, mental ill-health, dependence and death, although cardiovascular risks were a notable omission from young people’s responses. However, concern for the shorter-term or social consequences of alcohol consumption were also prominent and included: public drunkenness, damage to personal relationships, accidents and drink-driving. Some girls also stressed the possibility of gendered risks: sexual violence for girls and physical violence for boys.

C2 participants additionally drew upon their experiential knowledge of alcohol consumption to emphasise the undesirable effects it could have, including vomiting, anti-social behaviour and hangovers, all of which were argued to be deterrents to drinking. Henry, for example, asked: ‘Who wants to really throw up and have a hangover? I mean it’s not really worth it in my opinion’ (male, C2, University Student).

Different patterns of drinking were, however, thought to be associated with different levels of risk of harm. Binge drinking, or daily drinking, were considered the antitheses of safe drinking. Nevertheless, alcohol consumption was generally described as purposeful and oriented towards intoxication to some degree; ‘I only want to drink if I know I’m going to feel the effects’ (Charlotte, female, C2, urban affluent). In this context, ‘properly-managed’ drunkenness was seen as a positive outcome of drinking, associated with a level of risk of harm that young people were prepared to accept.

Among survey respondents, the potential for alcohol related harm was the most frequently asserted contributory factor to the decline in youth alcohol consumption (32%). This remained true when sub-dividing responses by age and gender. In both datasets, discussion of risk and the associated patterns of self-governance were explicitly linked with the desire to avoid health-related harms, rather than a decision to promote healthy lifestyle choices.

### Contemporary youth cultures and places of socialisation

Several aspects of contemporary youth culture were considered to be mediators of reductions in youth alcohol consumption by interview participants. In summary, these elements related to the timing and locations of young people’s alcohol consumption, and – as also suggested in the parenting theme below—a shift in the social cachet of alcohol for today’s young people relative to prior generations.

First, infrequent socialisation outside of the home during the school week and the importance of social media for everyday socialisation were highlighted. This was especially evident in the accounts of participants still in compulsory education and aged under 18. Moreover, there was also general agreement that young people benefited from the availability of diverse forms of distraction and entertainment – particularly, though not exclusively, associated with the use of social media—that both mitigated against boredom and facilitated forms of peer interaction within which alcohol played little, if any, part.

Second, legislative restrictions have significantly curtailed contemporary young people’s access to spaces licensed to sell alcohol and young people have responded with a shift away from public sites of alcohol-related socialisation, such as pubs. Drinking outside in parks or ‘the woods’ was also frowned upon and considered a mark of immaturity, or an unnecessary risk. Consequently, underage drinking was largely limited to parties within private homes, at weekends, or on special occasions. Drinking occasions were also timed to ensure they did not coincide with exams or impede study.

Third, alcohol was argued to have lost much of its potency as a marker of rebellion within youth culture: ‘you’ve not got the people trying to be like “Oh, I’m breaking the rules, I’m doing something I shouldn’t”, trying to get attention’ (Kelly, female, C2, rural). Young people across the age range noted both a lessening of pressure from peers to consume alcohol (compared with what they assumed had been the case previously) and a greater acceptance of abstinence. Nevertheless, alcohol retained value as a social lubricant for university students and there was a perception of other – and othered—manifestations of peer culture, within which alcohol consumption was more prevalent.

It probably varies a lot around the UK, […] where in some parts it’s kind of cool to go out drinking both days of the weekend like Friday, Saturday, Sunday, like, skipping school to go and get mashed or whatever, but then I kind of hear that’s just a bit desperate, like.

(George, male, C2, urban affluent)

Changes in youth cultures and in young people’s experiences and places of socialisation were the second most common theme in the survey data (27%). Within this theme, and consistent with the interview data, three sub-themes were identified. First, alcohol was described as no longer highly valued in youth culture, partly due to its ubiquity, and a resultant lack of ‘hype’ or ‘coolness’ around it: ‘It’s become almost normalised to drink alcohol as you grow up you realise it’s not as rebellious as it seems and it loses its thrill in a way’ (C1 female). Second, young people described themselves as being able to enjoy themselves without recourse to alcohol (‘They don’t need it to have fun’, C1 male). Third, relative to prior generations, young people described themselves as having less free time, as socialising less frequently, and as socialising in ways that did not require leaving the home and/or face-to-face interaction.

### The affordability of alcohol

Young people commonly referred to cost when discussing the decline in youth alcohol consumption. However, this played out quite differently among young people from different contexts. Young people in the affluent schools emphasised the *relative* cost of alcohol within a broader landscape of purchasing decisions. Although they had access to money to spend, they preferred to use this to purchase food, clothes, books or public transport rather than to buy alcohol:

Interviewer: What is it you choose to spend your money on then?

Tom: Going to town, snacks from Sainsbury’s [*major supermarket chain*] and stuff.

(male, C1, urban affluent school).

A few of the C2, affluent young people also reported saving their money for significant projects: university or to take holidays with friends. In contrast, young people from the deprived and rural schools, and from the FE colleges, indicated that they lacked adequate access to financial resources and the cost of alcohol was seen to be a deterrent to drinking, particularly when purchased in city centre locations. Maisie, for example, declared drinking in town to be: ‘More expensive, innit [isn’t it]’ (female, C1, rural school).

Whilst recognising that alcohol pricing might deter young people from some consumption practices, participants also pointed to taking alcohol from parental stores or purchasing cheap forms of alcohol as low-cost alternatives.

Cost was the third most commonly cited reason for the decline amongst survey responses (22%). In contrast to the interview data, where perspectives were differentiated most obviously by socio-economic status, survey responses referring to costs were differentiated by age. Reflecting the reality that most underage supply of alcohol is social [48], C2(29%) – not C1 (10%)—respondents tended to assert the high cost of alcohol as a plausible contributor to the decline in youth drinking.

### Displacement of alcohol by other substances

All references during interviews to the displacement of alcohol by other substances occurred in discussions with C2 participants, potentially reflecting the greater likelihood of older adolescents having direct or indirect experience of wider substance use. Faith (female, C2 further education college), for example, explained that ‘there’s other things like drugs. If they’re not drinking alcohol I feel like they might be doing drugs such as weed.’ Weed (cannabis) was the most frequently mentioned alternative substance in all educational contexts.

Displacement of alcohol by other drugs was considered to be driven by ease of access: ‘it’s just easier to get other things now than it is to get alcohol because you don’t need ID to go and buy some drugs off some random man, do you?’ (Annabelle, female, C2, rural). For those in the deprived school where limited financial resources were noted as a barrier to alcohol use, drugs could also offer a cheaper alternative: ‘obviously alcohol’s monitored, it’s taxed, things like that, but obviously you can’t go to the shop and buy Spice, meaning you’re going to get it for less.’ (Liam, male, C2, urban deprived).

Further, the relative risk of using cannabis, compared with consuming alcohol, was thought to be low: cannabis was argued to have ‘far more benefits than disadvantages’ (Charlotte, female, C2,urban affluent). Cannabis was felt to allow young people to experience a pleasurable ‘high’ without the risk of a hangover the following day and, for affluent young people, the consequent negative effects that this could have on school, university or other work. In this way, the displacement of alcohol by cannabis was framed as a positive choice – derived from young people’s future orientations toward their education *and* their health.

Reflecting interview responses, age was also an important intersection in the survey data. As noted earlier: 21% of C2 respondents cited drug substitution as a plausible driver of decline, but only 10% of C1 respondents did. Overall 17% of respondents suggested that the substitution of alcohol by other substances might be contributing to the decline in youth drinking. The ready availability and the low cost of alternative substances, in comparison with the cost of alcohol, was reaffirmed by survey respondents.

### Access and the regulatory environment

In interviews, young people across contexts and age cohorts, referred to a stricter regulatory environment as a reason for the decline in youth alcohol consumption. Participants cited family stories as key to their understandings of such changes. The penalties imposed on retailers who sell alcohol to young people were highlighted, in particular ‘Challenge / Think 25’ (a scheme that encourages retailers to check the identification of anyone who looks under 25 to prevent sales to under-18 s). Olivia, for example, noted:

Well I would have said it’s got a lot stricter, like, whenever I talk to my mum about it or my grandparents, like, my granny was talking the other day about it and she was saying that, you know, she’d go to the bar at fourteen and get served and not be questioned.

(Olivia, female, C2, university student).

Although increased regulation could make access to alcohol more difficult, young people described strategies used to mitigate the deterrent effect. These included buying or borrowing identification in order to purchase alcohol, or drawing on parents’ willingness to supply alcohol to young people.

Perhaps reflecting the ability of young people to bypass restrictions on their access to alcohol (or their lack of desire, or need to buy alcohol), the regulatory environment was only cited in 11% of survey responses. Stricter laws, more stringent enforcement, and policing of regulations, and increasing surveillance of licensed venues were nonetheless considered to be contributory factors to reductions in youth drinking within survey data.

### Disputing the decline

Few young people were aware of the decline in youth drinking. Indeed, a notable minority argued that alcohol remained highly visible in their everyday lives or peer relationships. The claim that drinking was in decline was therefore openly challenged by a number of participants from all educational contexts. Charlotte (female, C2, urban affluent school), for example, noted that: ‘I honestly don’t feel that there’s a visible decline, obviously we didn’t see our parents when they were teenagers, but I wouldn’t say we don’t drink a lot’.

The same scepticism was evident but less prominent in survey responses, being cited by only 9% of respondents. While the majority of such respondents simply denied that there had been a decline, with statements such as “They’re not [*drinking less*]”, some drew on personal experience to report heavy drinking either in their neighbourhood or amongst their peers. A small number of respondents suggested that that drinking had been masked, rather than reduced, by young people’s increasing skill in hiding their alcohol consumption from the adult gaze: “they’re just sneaky about it” (C1, girl) and are “doing it more secretly” (C2, boy).

### Future orientations

A number of C2 young people in the affluent schools described their generation as more mature and responsible than previous generations and more concerned about ‘doing well’ to secure their futures.

‘I think especially like in sixth form [*the final two years of secondary school education, typically ages 16-18*], like, it is a lot of work, we have like a lot of homework and everything and I feel like everyone wants to do good’

(Agnes, female, C2, urban affluent).

George and Alice (male and female, C2,urban affluent) also noted, they did not want to ‘throw away’ the opportunities that school offered them and young people in the affluent school were keen to achieve their academic potential and to study at university. School-related work was therefore an important structuring element in their everyday lives, as Iqbal (male, C2, urban affluent) made clear. He described spending ‘a few hours every day or something like that, just to like get homework done literally and keep up with notes, you’ve got to do a set amount’.

Focussing on educational achievement was seen as key to optimising young people’s futures and therefore as antithetical to drinking. As such, it was a plausible reason from the participants’ perspective for the decline in youth alcohol consumption.

Although ‘future orientations’ was amongst the least frequently cited reasons for the decline in the survey data (8%), a concern for young people’s future wellbeing was nonetheless evident. One young man (C1) noted: ‘We’re more worried about our futures than anyone so far – we don’t have the time or the privilege to waste getting drunk’. In contrast to the interview data where there was a predominance of data from C2 participants from the affluent school, evidence that concern for the future constituted a reason for declines in youth drinking emerged in the survey at a consistent level across age cohorts (8%); there was no socio-economic data to cross-reference. The aforementioned absence of data relating to ‘future orientations’ from specific subsets of young people within interviews cannot necessarily be interpreted as indicative of a difference in mind-set, however.

### Parenting and the home environment

Young people in interviews highlighted changes in parenting practices as significant for the decline in youth drinking. Nafeesa (female, C2, further education college), for example, suggested: ‘I think parents probably might be stricter and might have an influence’. Contemporary parents were described as more concerned about under-age drinking than previous generations of parents and were thought to maintain closer surveillance over their children. C1 young people asserted that their parents would not wish them to drink at all while, within C2 accounts, parents were noted to advocate *responsible* drinking practices.

With the exception of families with religious beliefs that did not allow the consumption of alcohol, the majority of young people noted the continuing presence – rather than the absence—of alcohol and its ready accessibility in their home environments. Young people were therefore often able to access alcohol in the home either without parental permission—by ‘sneaking’ a drink (Jessica, female, C1, urban deprived), or with parental permission as part of their induction into ‘responsible drinking’. For example, Kelly (female, C2, rural), noted that her ‘first taste’ of alcohol was provided by her mum, at home.

Similar issues were raised by survey respondents. Although changes in parenting were least commonly noted as potential contributors to the decline in youth drinking (6%), similar sub-themes were identifiable in the free-text data. Respondents described contemporary parents as ‘stricter’, more controlling or more protective. However, a small number suggested that rather than being more controlling, some parents were more lenient and permissive, normalising drinking ‘so it becomes less of a taboo’ (C1 female), and ‘Because our parents let us drink small amounts younger, therefore meaning that as we get older, the urge to drink is much less severe’ (C1, male).

## Discussion

The research identified eight key themes that young people used to explain the decline in youth drinking and these were largely consistent across the two phases of the research. The themes were: Disputing the decline; The affordability of alcohol; Access and the regulatory environment; Parenting and the home environment; Future Orientations; Displacement of alcohol by other substances; The potential for alcohol-related harm; and Contemporary youth cultures and places of socialisation. The survey data additionally demonstrated a degree of heterogeneity in the experiences and perspectives of different groups of young people, such that those of different ages, gender, and socio-economic position appeared more or less likely to identify particular explanations for the decline.

The key themes derived from young people’s perspectives resonated strongly with reasons for the decline of youth drinking that have been hypothesised in the academic literature. Building on this, we further describe the importance of place and the responsibilisation of youth in shaping contemporary youth drinking practices in England. While both the qualitative data and the survey responses addressed a range of factors thought to be contributing to the decline in youth alcohol consumption, changing notions of risk and the ways these are interwoven with shifting social contexts appear to underpin many of these. In some cases, risk related to young people’s perceptions of risks to themselves, while in other cases risk related more to others’ perceptions of risks associated with young people.

For example, respondents highlighted the stringent regulatory retail environment for alcohol and the difficulties that under-age consumers had in accessing alcohol from licensed vendors. Regulatory changes in the UK have, to some extent, been driven by the desire to prevent binge drinking and the crime and anti-social behaviour associated with it, and to deter underage drinking. This arises in part from the growing understanding of the potential and specific damages of alcohol to young people [49], as well as the perceived threat that disorderly behaviour by young people poses to wider society [50].

Partly as a result of these regulatory constraints on access to alcohol and also the high cost of alcohol in licensed premises [51], drinking was almost entirely limited to the home for today’s underage drinkers. Domestic spaces were also key sites for drinking and ‘pre-drinking’ by those aged 18 and over [52–54]. However, this ‘homification’ of young people’s alcohol consumption also arises from other non-risk related factors. In the UK context, for example, specific policies targeting anti-social behaviour have sought to problematise and diminish young people’s presence and visibility within outdoor public spaces (for example, [55]). Such policies, we argue, reflect well-established contentions that some young people are seen to be ‘risky’ in public spaces, while others are ‘at risk’ [56]. And as James ([38]:15) has noted, young people’s social embeddedness means that they are, inevitably, influenced by such cultural moralities and institutional constraints.

Young people highlighted that underage drinking has become less socially acceptable to contemporary youth. Both displays of public drunkenness (in digital or physical spaces) and drinking in outdoor spaces – a practice reported for previous generations of youth, were denounced. This was particularly true within affluent and Higher Education contexts, perhaps suggesting that the ‘sensible, low-risk drinking message’ advocated within the English National Alcohol Strategy 2007 [57] has perfused cultural sensibilities in at least some sub-groups of young people. In addition to their denouncement of risky drinking, however, participants also described diversification of young people’s leisure-time activities – often including the physically-distanced use of digital media and/or gaming – that have opened up opportunities for young people to socialise in ways that do not involve alcohol consumption [30] and which, often, exclude the use of outdoor and public spaces.

Although evidence for changes in parenting styles over time is sparse [58], young people postulated that parenting had changed toward greater levels of strictness and surveillance. In general terms, this reaffirms accounts describing the intensification of contemporary parenting and greater levels of risk aversion amongst contemporary parents [59]. In terms of alcohol drinking specifically, it also echoes Larm et al.’s [58] finding that parental monitoring and restrictive parental attitudes toward alcohol have increased during the period of declining youth drinking. Moreover, some young people in our study suggested a more complex relationship between changes in parenting and trends in youth drinking, perhaps reflecting the diversities in familial adult–child relations, characterised by Zeiher [40] as dependent, independent and interdependent. Young people in our study argued that it is the relative *leniency* of contemporary parenting that may support declines in drinking by reducing the need for young people to rebel against parental rules. Nevertheless, findings in the broader alcohol literature suggest that parents harbour concerns that ‘strictness’ in relation to alcohol consumption can be counter-productive [6, 60–62]. Any association between particular parenting styles and the emergence of recent alcohol trends is therefore unclear.

Young people’s suggestion that they exhibit greater maturity and responsibility than previous generations was closely aligned to how they made sense of the ‘risks’ posed by uncertain futures [63]. Educational success was considered key to some young people’s accomplishment within an increasingly competitive global economy. Consequently, young people restricted their drinking occasions to time periods when alcohol consumption would not impede their attainment. However, an important caveat to this finding is that a number of interview respondents were at a time in their education where they were due to sit national examinations and this may have sharpened the importance of educational success in their mindsThis finding aligns with the reduced frequency of alcohol consumption amongst 11–24 year olds in England [4]. The lack of references to the importance of education amongst deprived interview respondents, however, potentially reaffirms the centrality of class as a critical factor in the future economic security, and the educational experiences and aspirations of today’s young people [50]. It also confirms and reinforces the need to understand how perspectives and expectations, as they relate to alcohol consumption, manifest for individual young people who are variably located with respect to such issues as class, ethnicity, gender and age.

While the UK Office for National Statistics (ONS) [64] has described today’s young people as ‘Generation Sensible’ because they’shun alcohol, tobacco and even sex’, declines in youth drinking have also been accompanied by declines in youth drug taking in England [42]. Despite this apparent risk aversion, young people in our study – particularly those aged-16–18 for whom drugs were visible in their everyday lives—considered drug substitution a plausible reason for declines in alcohol consumption. They emphasised cannabis as a key substitute for alcohol, cohering with data demonstrating that this is the most commonly used drug amongst young people [65] and reinforcing the suggestion that polices designed to limit alcohol use may have the unintended consequences of increasing cannabis use among some young people [66]. Cannabis was seen to be an accessible, low cost substitute for alcohol, echoing findings from a recent review by Subbaraman [67], which suggests that substitution is likely to occur in environments in which cannabis can be obtained with relative ease. However, importantly, substituting alcohol with cannabis was viewed as a risk-reducing activity. Our respondents argued cannabis has positive benefits for young people. They thought it was less harmful and less likely to be associated with negative effects on their school or work lives when compared to alcohol.

Alcohol–related harm was strongly associated, by young people, with notions of binge drinking [68]. In our study, young people sought to distance themselves from the notion of binge drinking and from the pejorative associations between youth and excessive (‘morally questionable, unregulated’ ([69]:154)) alcohol consumption that have been the subject of sustained media and political condemnation [70–73]. Young people sought to distance their own ‘unproblematic’ or ‘social drinking’ from problematic ‘binge drinking’. This distinction, which drew upon ‘subjective personal assessment(s) of drunkenness’ ([73]:212) was well illustrated by one participant, Greg, who noted that 6 or 8 units of alcohol consumed over a short period of time would be considered to be binge drinking [73]. Yet, his own consumption of ‘maybe 3 pints’ over a couple of hours was deemed acceptable, despite the equivalence in terms of units of alcohol (Greg, male, C2, University Student) [57]. Our findings therefore further illustrate the importance that contemporary young people attribute to self-governance, individualised management of risks and deliberative and controlled attitudes to pleasure [37, 69, 74, 75]. They also highlight that risks assumed particular significance in light of young people’s future-oriented concerns and were intentionally moderated in light of such concerns.

## Strengths

This study’s young-person-centred approach provides important, contextualised understandings of the decline in youth drinking and, through this, extends the extant academic and policy literature. Additionally, interview data was generated with a diverse sample. Close alignment between the questions asked in the survey and the interview allows critical comparison between the two datasets. This is a novel approach in this field.

Our commitment to a child and young person centred approach throughout the research process enabled the active participation of young people in data generation, supported young people’s agency and demonstrated the extent to which individual young people’s expressions of their agency are inevitably influenced by their social context [76]: as Smart [77] has noted, young people’s lives are characterised by ‘”connectedness and embeddedness in and with the social and the cultural” ([77]:188). This interconnectedness, and young people’s embeddedness within particular social and cultural contexts, is reflected in our findings, providing a reminder of the need to recognise diversity between young people, as well as the commonality within the themes presented in this paper.

## Limitations

A number of limitations to this study need to be acknowledged. While this paper presents findings from England, all data were generated with 12–19 year- olds in one post-industrial northern city. The potential for national, regional and/or local variability and amongst young people up to the age of 24 (who have also shown declining consumption) merits exploration. Further, recruitment of rural schools was challenging. The catchment areas of the rural schools that were recruited included young people living in rural, peri-urban and urban areas who were also relatively, socio-economically deprived (as assessed by proportion of Free School Meal recipients). Moreover, we were unable to recruit young people who were not participating in formal education (those not in employment, education or training – often referred to as NEETs), despite identifying the importance of understanding their perspectives.

While the sample for phase one of the study was ethnically and socioeconomically diverse we did not collect data regarding ethnicity, socioeconomic position or urban/rural location for our phase two participants. The relative diversity of survey respondents is therefore unknown. Female and C2 survey respondents also outnumbered male and C1 respondents. Interpretation of the frequencies of responses by theme should be viewed in light of this imbalance, although the purpose of the survey was to provide indicative evidence of the prevalence of different perspectives rather than representative population data.

A further important limitation was associated with the need to curtail interviews in schools and colleges when students had to move to their next lesson, meaning that not all participants were asked to speculate on reasons for the decline.

### Implications for policy and practice

This paper demonstrates the importance of understanding alcohol consumption within the context of broader youth cultures and consumption practices, as well as wider public policy affecting youth [78]. Through this, the salience of risk in shaping youth behaviours is highlighted. Moreover, this paper emphasises the necessity of approaches to health promotion and protection that do not attribute primacy to one factor, but appreciate the interconnections and overlap between individual drivers of health trends.

Understanding young people’s own perspectives on factors contributing to the decline in alcohol consumption could help to sensitive and optimise public health policies aimed at young people in the future, for as James ([38]:174)points out, what young people do or say “can have an effect on other people, ideas, events and also on the ways in which policy interventions in their lives take root or not”.

### Implications for research

The reasons highlighted by young people as drivers of declines in youth alcohol drinking may be more or less salient for different groups. Although our analyses highlight gendered, age-based and socioeconomic divergences in the data, particularly in relation to affordability, other substance use, further enquiry in relation to these intersections and urbanity/rurality would be of value. Additional qualitative enquiry with older age cohorts would complement and permit further interrogation of the assumptions young people in this study make about changes in youth culture over time and the experiences of prior generations of young people. 

## Conclusion

This paper highlights the commonality between the reasons young people assert and those given attention in the academic and policy literature. Importantly, the divergent ways in which declines in youth drinking are understood and experienced between different groups of young people. We conclude that there is a perceived cultural shift between generations of young people, manifesting in terms of their spaces and places of socialisation, and shifting attitudes towards risk, self-governance and the future.

FE (Further Education) colleges provide educational opportunities for students aged 16 or over in England. Students usually follow courses that are necessary to progress into Higher Education (the University sector), or into a specific career path.

## Supplementary Information

**Additional file 1:** **SupplementaryTable 1.** Sample characteristics; interview participants. **Supplementary Table 2.** Themes; frequency of responses by cohort and gender.

## Data Availability

The datasets used and/or analysed during the current study are available in anonymised format from the corresponding author on reasonable request.

## Abbreviations

NEET: Not in employment, education or training
C1: Cohort One
C2: Cohort Two
UK: United Kingdom
