# Supplementary material for: Young people’s explanations for the decline in youth drinking in England
Source: BMC Public Health. 2023 Feb 28;23:402. doi: 10.1186/s12889-022-14760-y (PMC9972726; doi:10.1186/s12889-022-14760-y)
Supplement: Supplementary file 1 — Additional file 1: SupplementaryTable 1. Sample characteristics; interview participants. Supplementary Table 2. Themes; frequency of responses by cohort and gender. [file 12889_2022_14760_MOESM1_ESM.docx]

# Title Page

**Young people’s explanations for the decline in youth drinking in England.**

*Victoria Whitaker^2^, Penny Curtis^1^, Hannah Fairbrother^1^, Melissa Oldham^3^, & John Holmes^2^*

*^1^Health Sciences School, University of Sheffield, Sheffield, UK*

*^2^School of Health and Related Research, University of Sheffield, Sheffield, UK.*

*^3^ Behavioural Sciences and Health, University College London, London, UK*

**Corresponding author: Dr Victoria Whitaker,** [**v.whitaker@sheffield.ac.uk**](mailto:v.whitaker@sheffield.ac.uk)

### Supplementary Table 1: Sample characteristics; interview participants

|  | **Affluent schools** | | **Deprived school** | | **Rural schools** | | **FE Colleges** | | **University Students** | | **Total** |
| --- | --- | --- | --- | --- | --- | --- | --- | --- | --- | --- | --- |
|  | Boys | Girls | Boys | Girls | Boys | Girls | Boys | Girls | Boys | Girls |  |
| **Number of participants** |  | | | | | | | | | | |
| Cohort One | 8 | 5 | 6 | 9 | 7 | 8 |  | | | | 43 |
| Cohort Two | 7 | 7 | 5 | 5 | 8 | 8 | 3 | 3 | 2 | 5 | 53 |
| **Age of participants** |  | | | | | | | | | | |
| 12 years |  |  |  |  | 4 | - |  | | | | 4 |
| 13 years | 7 | 2 | 3 | 7 | 3 | - |  |  |  |  | 22 |
| 14 years | 1 | 3 | 3 | 2 | - | - |  |  |  |  | 9 |
| 15 years | - | - | - | - | - | 8 |  |  |  |  | 8 |
| 16 years | 2 | 3 | 2 | 4 | 4 | 4 | 1 | - | - | - | 20 |
| 17 years | 3 | 1 | 2 | 1 | 3 | 2 | 2 | - | - | - | 14 |
| 18 years | 2 | 3 | 1 | - | 1 | 2 | - | 3 | - | 2 | 14 |
| 19 years | - | - | - | - | - | - | - | - | 2 | 3 | 5 |
| **Ethnicity** |  | |  | | | | | |  | |  |
| White British | 10 | 8 | 6 | 7 | 14 | 16 | 3 | - | 1 | 4 | 69 |
| Other White Background | - | 2 | - | 1 | - | - | - | - | 1 | - | 4 |
| Mixed White and Asian | 2 | 1 | 1 | - | - | - | - | - | - | 1 | 5 |
| Asian British  (Bangladeshi) | - | 1 | 2 | - | - | - | - | - | - | - | 3 |
| Asian British (Pakistani) | - | - | - | 1 | 1 | - | - | 1 | - | - | 3 |
| Asian (Pakistani) | 2 | - | - | - | - | - | - | - | - | - | 2 |
| Mixed White and Black British | 1 | - | - | - | - | - | - | - | - | - | 1 |
| Mixed White and Black Caribbean | - | - | - | 1 | - | - | - | - | - | - | 1 |
| Black British  (African) | - | - | 1 | 1 | - | - | - | 2 | - | - | 4 |
| Black Caribbean | - | - | - | 1 | - | - | - | - | - | - | 1 |
| Black African | - | - | 1 | 2 | - | - | - | - | - | - | 3 |

### Supplementary Table 2: Themes; frequency of responses by cohort and gender

| Disputing the decline | | | | | | |
| --- | --- | --- | --- | --- | --- | --- |
|  | Male count | Male % | Female count | Female % | Total count | Total % |
| C1 | 6 | 7% | 16 | 12% | 22 | 11% |
| C2 | 17 | 14% | 10 | 5% | 27 | 8% |
| Overall | 23 | 12% | 26 | 8% | 49 | 9% |
| The affordability of alcohol | | | | | | |
|  | Male count | Male % | Female count | Female % | Total count | Total % |
| C1 | 9 | 12% | 11 | 9% | 20 | 10% |
| C2 | 35 | 29% | 63 | 30% | 98 | 29% |
| Overall | 44 | 22% | 74 | 22% | 118 | 22% |
| Access and the regulatory environment | | | | | | |
|  | Male count | Male % | Female count | Female % | Total count | Total % |
| C1 | 9 | 12% | 11 | 9% | 20 | 10% |
| C2 | 10 | 8% | 27 | 13% | 37 | 11% |
| Overall | 19 | 10% | 38 | 11% | 57 | 11% |
| Parenting and the home environment | | | | | | |
|  | Male count | Male % | Female count | Female % | Total count | Total % |
| C1 | 4 | 5% | 9 | 9% | 13 | 6% |
| C2 | 3 | 2% | 14 | 7% | 17 | 5% |
| Overall | 7 | 6% | 23 | 7% | 30 | 6% |
| Future orientations | | | | | | |
|  | Male count | Male % | Female count | Female % | Total count | Total % |
| C1 | 4 | 5% | 12 | 9% | 16 | 8% |
| C2 | 7 | 6% | 20 | 9% | 27 | 8% |
| Overall | 11 | 6% | 32 | 9% | 43 | 8% |
| Displacement of alcohol by other substances | | | | | | |
|  | Male count | Male % | Female count | Female % | Total count | Total % |
| C1 | 9 | 12% | 12 | 9% | 21 | 10% |
| C2 | 27 | 22% | 43 | 20% | 70 | 21% |
| Overall | 36 | 18% | 55 | 16% | 91 | 17% |
| The potential for alcohol related harm | | | | | | |
|  | Male count | Male % | Female count | Female % | Total count | Total % |
| C1 | 27 | 35% | 49 | 38% | 76 | 37% |
| C2 | 28 | 23% | 71 | 33% | 99 | 30% |
| Overall | 55 | 28% | 120 | 35% | 175 | 32% |
| Contemporary youth cultures and places of socialisation | | | | | | |
|  | Male count | Male % | Female count | Female % | Total count | Total % |
| C1 | 19 | 24% | 40 | 31% | 59 | 29% |
| C2 | 30 | 25% | 55 | 26% | 85 | 26% |
| Overall | 49 | 25% | 95 | 28% | 144 | 27% |
